# How to Better Understand the Influence of Host Genetics on Developing an Effective Immune Response to Thoracic Cancers

**DOI:** 10.3389/fonc.2021.679609

**Published:** 2021-06-21

**Authors:** Kiarash Behrouzfar, Kimberley Burton, Steve E. Mutsaers, Grant Morahan, Richard A. Lake, Scott A. Fisher

**Affiliations:** ^1^ National Centre for Asbestos Related Diseases (NCARD), University of Western Australia, Nedlands, WA, Australia; ^2^ School of Biomedical Sciences, University of Western Australia, Nedlands, WA, Australia; ^3^ Institute for Respiratory Health, University of Western Australia, Nedlands, WA, Australia; ^4^ Centre for Diabetes Research, Harry Perkins Institute of Medical Research, Nedlands, WA, Australia

**Keywords:** thoracic malignancies, tumor immune microenvironment, mesothelioma, Collaborative Cross, MexTAg, host genetics

## Abstract

Thoracic cancers pose a significant global health burden. Immune checkpoint blockade therapies have improved treatment outcomes, but durable responses remain limited. Understanding how the host immune system interacts with a developing tumor is essential for the rational development of improved treatments for thoracic malignancies. Recent technical advances have improved our understanding of the mutational burden of cancer cells and changes in cancer-specific gene expression, providing a detailed understanding of the complex biology underpinning tumor-host interactions. While there has been much focus on the genetic alterations associated with cancer cells and how they may impact treatment outcomes, how host genetics affects cancer development is also critical and will greatly determine treatment response. Genome-wide association studies (GWAS) have identified genetic variants associated with cancer predisposition. This approach has successfully identified host genetic risk factors associated with common thoracic cancers like lung cancer, but is less effective for rare cancers like malignant mesothelioma. To assess how host genetics impacts rare thoracic cancers, we used the Collaborative Cross (CC); a powerful murine genetic resource designed to maximize genetic diversity and rapidly identify genes associated with any biological trait. We are using the CC in conjunction with our asbestos-induced MexTAg mouse model, to identify host genes associated with mesothelioma development. Once genes that moderate tumor development and progression are known, human homologues can be identified and human datasets interrogated to validate their association with disease outcome. Furthermore, our CC−MexTAg animal model enables in-depth study of the tumor microenvironment, allowing the correlation of immune cell infiltration and gene expression signatures with disease development. This strategy provides a detailed picture of the underlying biological pathways associated with mesothelioma susceptibility and progression; knowledge that is crucial for the rational development of new diagnostic and therapeutic strategies. Here we discuss the influence of host genetics on developing an effective immune response to thoracic cancers. We highlight current knowledge gaps, and with a focus on mesothelioma, describe the development and application of the CC-MexTAg to overcome limitations and illustrate how the knowledge gained from this unique study will inform the rational design of future treatments of mesothelioma.

## Introduction

Thoracic cancers including lung cancer (LC), malignant mesothelioma and thymic epithelial tumors (TETs) are among the most lethal cancers (1). In addition to conventional treatment options for thoracic cancers such as surgery, chemotherapy and radiotherapy, immune based treatments including immune checkpoint therapies, have improved treatment outcome for some patients (2, 3).

Cancer immunotherapy aims to restore or enhance the host’s immune system to recognize and eliminate cancer cells (4). Although immunotherapies have improved treatment outcomes for some thoracic cancers, success is often limited to a subset of patients, while prognosis for the majority of patients remains dismal (5). This dichotomy in response, highlights the need to better understand interactions between thoracic cancer cells and the host immune system that underpin an effective response to cancer immunotherapy.

Advances in high-throughput sequencing technologies and associated computational analysis pipelines allow us to investigate the interplay between tumor cells and the immune microenvironment (6, 7). These technologies enable us to broaden our knowledge of the immunobiology of tumor-host interactions by identifying immune-related genetic alterations associated with cancer development (6, 7). While genetic alterations associated with immune response in thoracic cancers have been exploited to improve treatment outcome (8, 9), the development of strong, durable responses occurs in a limited subset of patients (10); further highlighting the importance of understanding the role of host genetics, in addition to tumor genetics, in thoracic cancer development, for predicting response to immunotherapies (9, 11–13).

In this review, we discuss how host genetics affects the development of an effective immune response to thoracic cancers. We highlight knowledge gaps in our current understanding and acknowledge the limitations related to identifying host genetic factors associated with thoracic cancer susceptibility and development of effective anti-tumor immunity. Finally, we propose our unique murine model; the MexTAg Collaborative Cross (CC−MexTAg), as a strategy to overcome current limitations of conventional genetic studies in mesothelioma, to improve our knowledge about the impact of host genetics on initiating immune responses and the developing tumor microenvironment.

## Host Genetic Factors and Thoracic Cancer Susceptibility

To date, many rare, high penetrance genetic variants such as *BRCA1, BRCA2, TP53, APC, and PTEN* have been associated with a genetic predisposition to cancer (14–16). However, these genetic alterations only account for a small proportion of heritable cancer genetic risk variants (14, 15). In fact, the combination of genetic variation in common low penetrance alleles and rare moderate-risk alleles has been recognized as the major genetic contributors to heritable cancer genetic predisposition (17–19).

Common low-penetrance genetic variants, including single-nucleotide polymorphisms (SNPs), have been identified by GWAS (20). These genetic studies determine the frequency of SNPs in patients compared to healthy individuals (20). More than 450 genetic variants associated with increased cancer risk for breast, prostate, colorectal and lung cancer have been identified; supporting the polygenic pattern of susceptibility in these cancers (18).

### Lung Cancer

Lung cancer is the most prevalent thoracic cancer, and chromosomal positions 15q25, 5p15.33 and 6p21 have been identified as susceptibility loci (21, 22). However, whether 15q25 is truly an independent susceptibility locus for lung cancer remains contentious, as genetic variants of nicotinic acetylcholine receptor (CHRNA) genes, which have been strongly associated with nicotine dependence and smoking behavior (23–26), are also present at this loci. Furthermore, genetic variants in 15q25 are mainly frequent in European populations and not Asian populations (23). Other independent susceptibility loci, 6p21 and 5p15, show significant levels of genetic polymorphisms associated with lung cancer risk in Asian populations, including Japanese and Korean. However, there are different risk variants within 6p21 locus observed between Asian and European populations (27).

A large meta-analysis of GWAS on Chinese and European populations identified 19 susceptibility loci significantly associated with non-small cell lung cancer (NSCLC) risk (28). Using identified genetic factors, this study proposed the polygenic risk score (PRS) strategy as an effective risk indicator of lung cancer, independent from age and smoking pack-year (28). However, the utility of the PRS strategy may be limited, as it was only used to predict lung cancer risk among the Chinese population, not in other cohorts comprised of different ethnicity and effect size for genetic variants (28). Furthermore, most genetic variants were only associated with a small improvement in the prediction of lung cancer risk, and were not any greater than major risk factors such as smoking and age (29–31).

### Malignant Mesothelioma

Malignant mesothelioma is a relatively rare thoracic cancer, inextricably linked to asbestos exposure. The relatively low number of samples available for study means that conventional genetic studies are often underpowered (1). Consequently, despite using separate and well−characterized cohorts of control and mesothelioma patients, numerous GWAS studies have failed to identify common genetic risk factors that can be considered broadly associated with mesothelioma (32–34).

Germline mutations in *BAP1* and some DNA repair genes have been considered as predisposing genetic factors associated with mesothelioma development (34–36). However, these genetic risk factors are not specific for mesothelioma alone and can predispose people to other cancers such as uveal melanoma (37).

### Thymic Epithelial Tumors

Thymic epithelial tumors (TETs) are rare thoracic cancers arising from epithelial cells of the thymus, and can be categorized as either thymomas or thymic carcinomas (38). Our current knowledge of the etiology and genomic alterations of TETs remains limited, and like mesothelioma, the small number of patients available for study often restricts the power of conventional genetic analyses (39, 40). Although we could not find any published GWAS associated with any form of TETs, Wang et. al., identified mutated *TP53* as the most frequent genetic alteration in TET patients (40). The authors used comparative sequence analysis to show a higher mutation incidence in epigenetic regulatory genes in thymic carcinoma compared to thymoma patients (40). Additionally, a study by Cortes–Ledesma et. al., demonstrated a strong causal relationship between the loss of the highly-specialized DNA repair enzyme tyrosyl-DNA phosphodiesterase 2 (*TDP2*) and increased thymic-derived cancer predisposition in ataxia telangiectasia affected individuals (41).

## The Importance of Host Genetics in Cancer Susceptibility: Shaping of the Tumor Immune Microenvironment

The tumor microenvironment (TME) consists of a variety of immune cells, endothelial cells, fibroblasts and associated tissue cells, and develops in part from the dynamic interactions between the developing tumor and the surrounding host tissue (42). One way to describe how host-tumor interactions play a significant role in shaping the tumor microenvironment, is through the impact on ‘field effect’ around the tumor (43). In the context of cancer development, the term ‘field effect’ refers to pre-neoplastic cellular and molecular changes that arise as a consequence of long−term exposure to environmental carcinogens in morphologically healthy tissues, promoting a ‘field of susceptibility’ to neoplasia initiation and progression (44). For instance, the presence of a high burden and pervasive positive selection of somatic driver mutations has been identified in normal human skin (45). In a cohort of patients undergoing blepharoplasty, positively selected ‘driver’ mutations were found in 18−32% of normal skin cells taken from 234 biopsies of sun-exposed eyelid epidermis. These data suggest that the frequency of driver mutations in physiologically normal skin cells is surprisingly high, with multiple driver mutations in cancer associated genes found in many ‘normal’ cells that had not yet acquired malignant potential. These findings raise the question as to what combination of intrinsic (additional mutations) or extrinsic (host genetics/anti−tumor immunity) changes are required for cellular transformation to proceed?

In addition to driver mutations, epigenetic alterations including DNA methylation and histone modifications can play a role in establishing a field effect contributing to cancer development (46, 47). A number of studies have indicated the influence of an epigenetic field effect around the tumor by identifying aberrant DNA methylation profiles in both tumor and normal adjacent tissues (48–52).

## The Effect of Known Host Genetic Factors on Tumor Immune Microenvironment of Thoracic Cancers

The influence of host genetics on the development of thoracic cancers remains poorly understood. Shen et. al., performed enrichment analysis of GWAS data to identify shared genomic regions and pathways between host genetic variants and somatic mutations in lung cancer (53). They identified an association between the SNP rs36600 at 22q12.2 and somatic mutations within *ARID1A* (53), a member of the SWI/SNF chromatin remodeling complexes associated with many cancers (54). Mutations in *ARID1A* and *ARID1B* are also associated with improved response to NSCLC patients receiving immune checkpoint blockade (ICB) therapy (55). Elevation of the tumor mutational burden, enhanced antigen presentation and cellular immunity, and increased PD-L1 expression, are all correlated with the presence of ARID1A and ARID1B mutations; suggesting that mutated ARID1A and ARID1B could serve as novel biomarkers to predict sensitivity and prognosis to ICB in advanced NSCLC patients (55). Additionally, rare missense variants in genes encoding SWI/SNF chromatin remodeling components and genes encoding the histone methyl transferases, *SETD2* and *SETDB1*, were identified in a cohort of Japanese mesothelioma patients (56).

Furthermore, *TRB*:rs1964986 and *IDO1*:rs10108662 have been identified as the two most significant SNPs associated with the risk of disease recurrence and death respectively in early stage lung cancer (57). When assessing the functionality of T cells between low and high-risk groups relative to healthy controls (57), high-risk subjects exhibited lower cytotoxicity and reduced granulation of T cells, as demonstrated by increased expression of T cell inhibitory checkpoint gene Indoleamine 2, 3-dioxygenase (*IDO1*) and decreased expression of the T cell cytotoxicity genes *IL2*, Perforin 1 (*PRF*) and Granzyme B (*GZMB*). These data support the hypothesis that mutations of host immune genes affect the TME and thus, prognosis of NSCLC *via* suppression of T cell antitumor immunity (57).

Additionally, epigenetic alterations of tissues derived from NSCLC patients revealed the upregulation of *CTLA4*, *PDCD1 via* hypomethylation in tumors *versus* non-tumor tissues (58). Effects of epigenetic alterations in shaping immune tumor microenvironment was also demonstrated by strong correlation between site-specific DNA methylation of CpG markers of cancers and transcription of genes associated with immune infiltration (59).

In contrast to NSCLC, there are limited published studies that investigate the role of host genetic factors in shaping the tumor immune microenvironment of mesothelioma and TETs. Costa et. al., identified lower expression of miR-320 in mesothelioma tumors compared to normal tissues by performing differential miRNA expression analysis on 14 formalin-fixed paraffin-embedded tumors and six normal controls (60). They also identified an association between p53-induced expression of miR-320, miR-200a and miR-34a with reduced expression of *PD-L1* in mesothelioma cell lines (60). These data indicate defective p53-induced miRNA response as a possible contributor to immune evasion in mesothelioma by increasing tumor *PD-L1* expression (60). Reduced expression of major histocompatibility complex (*MHC*) and autoimmune regulator (*AIRE*) genes has been associated with defective T cell maturation in thymoma patients (61, 62) and as such, the reduced expression of *MHC* and *AIRE* were proposed as genetic alterations explaining the association between thymomas and autoimmune disorders (61).

More recently, a number of studies have significantly advanced our understanding of the molecular biology of mesothelioma (63–67). Through the application of next generation sequencing technologies and innovative bioinformatic analyses, these studies have demonstrated the complex heterogeneity within and between tumors; expanding the classic epithelioid, biphasic and sarcomatoid paradigm to at least 4 distinct molecular subtypes, with a molecular gradient along the epithelial-to mesenchymal transition spectrum separating the two extreme epithelioid-like and mesenchymal-like groups (63, 65–67). Additionally, the NSG approach has further advanced our knowledge of the key mutational events associated with mesothelioma, linking a number of unique cancer signaling pathways with mesothelioma (64, 65). However, despite these advances, our understanding of how host genetics impacts mesothelioma onset remains underdeveloped.

In summary, studies investigating the role of immune related genetic factors in NSCLC have identified suggestive genetic variants capable of shaping the tumor immune microenvironment and affecting cellular immunity. However, there are a limited number of studies identifying immune related genetic factors in mesothelioma and TETs, highlighting the dearth in our knowledge of, and ability to identify how host genetic factors shape the immune tumor microenvironment of rare thoracic cancers.

## Overcoming Limitations for Identifying Host Genetic Factors Associated With Thoracic Cancers

GWAS have been used to identify susceptibility loci in common cancers such as breast, prostate, colorectal and lung cancer (22, 68), but they have been less effective for rare cancers including mesothelioma (32). In fact, the suitability of GWAS for rare cancers is often restricted by the relatively small number of patients available for studies; thus any identified genetic variants are often limited to the study cohort and not likely to have a significant influence on disease outcome (32, 34, 68–71). Furthermore, the absence of standardized protocols for collecting environmental exposure data in addition to the lack of accurate, consistent and defined phenotypic data to match with genomic information, are additional potential limitations for human genetic studies of thoracic cancers (72–74).

To overcome these limitations, mouse models that can faithfully mimic human cancer development, in a well-controlled and modulated environment are needed for identifying translatable host genetic variants (75). Moreover, such mouse models need to be sufficiently genetically diverse to maximize the chance of genetic polymorphisms associated with cancer development. The ideal mouse model would enable rapid identification of genes associated with cancer homologous to human genetic studies (7).

## Advancements in Recombinant Inbred Mouse Models for Host Genetic Studies of Thoracic Cancers

Recombinant inbred (RI) mice are generated by breeding two or more different mouse strains to genetic stability (76). Historically, RI mice have been used to identify genomic regions, referred to as quantitative trait loci (QTLs), that are associated with particular disease phenotypes (77). The main advantage to using RI mouse strains compared to classical simple (F2) cross breeding is their improved reproducibility due to the ability to test a phenotype in any number of individuals with the same defined genetic constitution (77). The use of RI mice allows unknown genes to be integrated into the known genetic map by comparing the inheritance pattern of an unknown gene or trait in a panel of RI strains with that of known markers. Therefore, these models have been widely used as a robust and rapid method of gene mapping of polygenic traits and diseases (77).

## Use of Classical and Traditional Recombinant Inbred Mouse Models

Classical mouse genetic studies identified *Kras2* as a major lung cancer susceptibility locus using the F2 progeny of A/J (susceptible) and C3H/He (resistant) mouse strains in a urethane-induced lung cancer model (78). Similarly, experiments involving the progeny of BALB/c and SWR/J mouse strains identified *Par2* and *Par4* as modifier loci that specifically affected tumor initiation, progression and lung tumor multiplicity (79). Additional, whole-genome linkage disequilibrium analysis on 25 inbred mouse strains identified 63 markers including *Kras* and *Pas1* loci, supporting the association of *Kras* loci with lung cancer susceptibility (80). Traditionally, bi-parental RI mouse strains were used for identifying susceptibility loci of diseases with polygenic pattern of inheritance (78, 81, 82). However, the usefulness of traditional bi-parental RI mouse strains in genetic studies is limited by the inherent low genetic diversity associated with using only two parental genomes, which have large ‘identical by descent’ (IBD) regions in which both parental strains have the same alleles (83). Such IBD regions are ‘blind spots’, having little or no variation, thus limiting the potential for gene mapping (84).

## The Collaborative Cross (CC)

The Collaborative cross (CC) is a powerful mouse genetic resource, comprising hundreds of independent RI mouse strains developed from eight founder strains selected to maximize genetic diversity (84–86). The CC harnesses 90% of the common allelic diversity of the entire mouse species (84) and has enhanced mapping ability due to the much greater degree of polymorphisms derived from the eight diverse founder strains, rather than the two somewhat similar strains used in conventional RI mapping, as well as the greater number of strains available. Conventional mapping of simple Mendelian traits requires approximately 100 backcrossed mice to obtain 1 cM (approximately 2 megabase pairs; Mbp) resolution. The same resolution can be obtained by testing ~26 BXD RI strains. In contrast, with as few as 70 CC strains, mapping resolution can be less than 40 thousand bp, i.e. approximately to the single gene level (85) and even achieve down-to-the-base resolution (87). Thus, the CC allows mapping of loci with unprecedented accuracy. The CC has been successfully used to study diseases with polygenic inheritance such as melanoma, prostate cancer, diabetes and osteoporosis (86, 88–91). It is also powerful in allowing development of novel disease models (92).

The application of the CC to understanding cancer has been best studied for melanoma and skin cancer. A series of investigations made several important discoveries, such as that every stage of melanoma progression was subject to control by genetic variation (88); that UV−induced and spontaneous cancers were mediated by different genetic mechanisms (93) the specific mutation causing nevus development was identified (94); and the molecular mechanism for giant congenital nevi was defined (95).

## Using Genetically Engineered Mouse Models for Studying the Impact of Host Genetics on Thoracic Cancer Development

Numerous genetically engineered mice models have been developed for modelling and studying genetic heterogeneity of human malignancies (96). Recent technical advances in the manipulation and sequencing of mouse genomes has promoted the use of mouse models as an experimentally tractable system for testing hypotheses generated from human genetic studies (96). Such engineered models have also allowed the identification of novel candidate mechanisms linking the impact of host genetics and cancer development (96, 97). As the development and use of genetically engineered mouse models is time-consuming and expensive, the generation of models with high tumor penetrance and short cancer latency are often favored, as they are more viable in terms of research time and cost (75, 98).

*Kras2^LA2^* and *Trp53* ^LSL-R172H /þ^ mice are the most common used models in host genetic studies of lung cancer (96, 99, 100). However, mice with mutations in *Kras2* and *Trp53* are highly predisposed to other cancers (101, 102) and cancer development can be triggered by spontaneous oncogene recombination events; thus these models are not necessarily lung cancer specific and therefore some mechanisms of carcinogenesis may not accurately recapitulate human disease (99, 100).

There are many excellent mouse models for mesothelioma research that mimic the genetic defects found in human disease. Knockout (KO) mouse models with heterozygous mutations in *Bap1, CDKN2A*, neurofibromin 2 (*Nf2*), or *p53* have been used to study the effect of genetic alterations on asbestos-induced mesothelioma susceptibility (103–112). These studies demonstrate significantly higher incidence of mesothelioma in the presence, or absence of asbestos in mice with *Bap^(+/-)^* and *Nf2^(+/-)^* mutations compared to wild-type (wt) mice (103–112). However, all animal models have their limitations. While some conditional KO models demonstrate increased mesothelioma incidence, they also have moderate to high levels of unrelated (non-mesothelioma) cancers; presumable a consequence of off-target deletion of key tumor suppressor genes (105, 108, 110). However, recently published CRE-mediated conditional KO models only develop disease in CRE-expressing tissues (109, 113). Mesothelioma incidence in *Bap1^(+\-)^* mice ranged between 36-60% depending on asbestos doses (41, 106). However, while *Bap1^(+\-)^* mice develop mesothelioma, they also develop other cancers such as uveal and cutaneous melanoma (37). Furthermore, *Bap1^(+\-)^* mice develop mesothelioma after exposure to doses of asbestos fibers that are unlikely to induce mesothelioma in wt mice (106).

## Asbestos Induced Mesothelioma MexTAg Mouse Model

There is no absolute consensus over which is the best model system for studying mesothelioma; some groups prefer to use conditional knockout models, as they replicate the genetic deletions observed in human disease, while others prefer alternative models that require asbestos induction and have limited unrelated cancer development. We developed the transgenic C57BL/6 MexTAg mouse model expressing SV40 large T antigen directed to mesothelial cells by use of cell-type specific mesothelin promoter as a tool for the pre-clinical evaluation of asbestos-induced mesothelioma (114). Importantly, MexTAg mice develop mesothelioma with similar pathology to humans, but only after asbestos exposure (115). Furthermore, MexTAg mice have high disease incidence (> 85%) and are less likely to develop unrelated tumors compared to wild type mice or some heterozygous or conditional knockout models (103–112). Comparing gene expression profiles of MexTAg mice and wt mesothelioma with their counterpart normal mesothelial cells, exhibits overlapping gene expression profiles, suggesting a similar overall mechanism of mesothelioma development in transgenic MexTAg mice (116). Expression of the TAg transgene does not affect the overall mechanism of mesothelioma development, but rather phenocopies *p16* loss (117) and as a consequence onset of disease is more rapid, significantly increasing the incidence and rate of mesothelioma development compared to wt mice (114).

## Using the CC-MexTAg Mouse Model to Assess the Impact of Host Genetics on the Developing Anti-Tumor Immune Response to Mesothelioma

To investigate how host genetics might impact asbestos related disease development (ARD), it is important to use a model in which only ARD (and not unrelated tumors) occur and high incidence. We developed the MexTAg Collaborative Cross to investigate how a hosts’ genetic background influences the development of mesothelioma in asbestos-exposed individuals. Combining the genetic diversity inherent in the CC with the high incidence of asbestos−induced disease and rare onset of unrelated spontaneous tumors of MexTAg mice, provides an ideal model to define with unprecedented accuracy the genes and associated pathways that affect susceptibility and resistance to disease. In this model, the F1 progeny of CC x MexTAg mice (CC−MexTAg mice) are exposed to asbestos and monitored for up to 18 months, or until asbestos related disease (ARD) developed and progressed to a clearly defined endpoint. ARD phenotypic traits such as overall survival, disease latency and progression for each CC−MexTAg group, can be analyzed using the GeneMiner™ bioinformatic portal (87), where candidate modifier genes are mapped with ARD phenotype as a quantitative trait. Genome wide scans defined chromosomal locations of peak SNPs associated with each of the characterized ARD phenotypes. To date, we have generated and asbestos-exposed over 2500 individual CC−MexTAg mice progeny of 72 unique CC strains. At the time of writing 55 CC−MexTAg groups that have completed the observation period. These preliminary data indicate greater than 3-fold variation in median overall survival. This shows the power of the CC approach, given that the parental MexTAg mice survive 365 days. An additional 20 CC−MexTAg groups remain under study, and we envisage accrual of complete data by late 2021.

**Figure 1:**
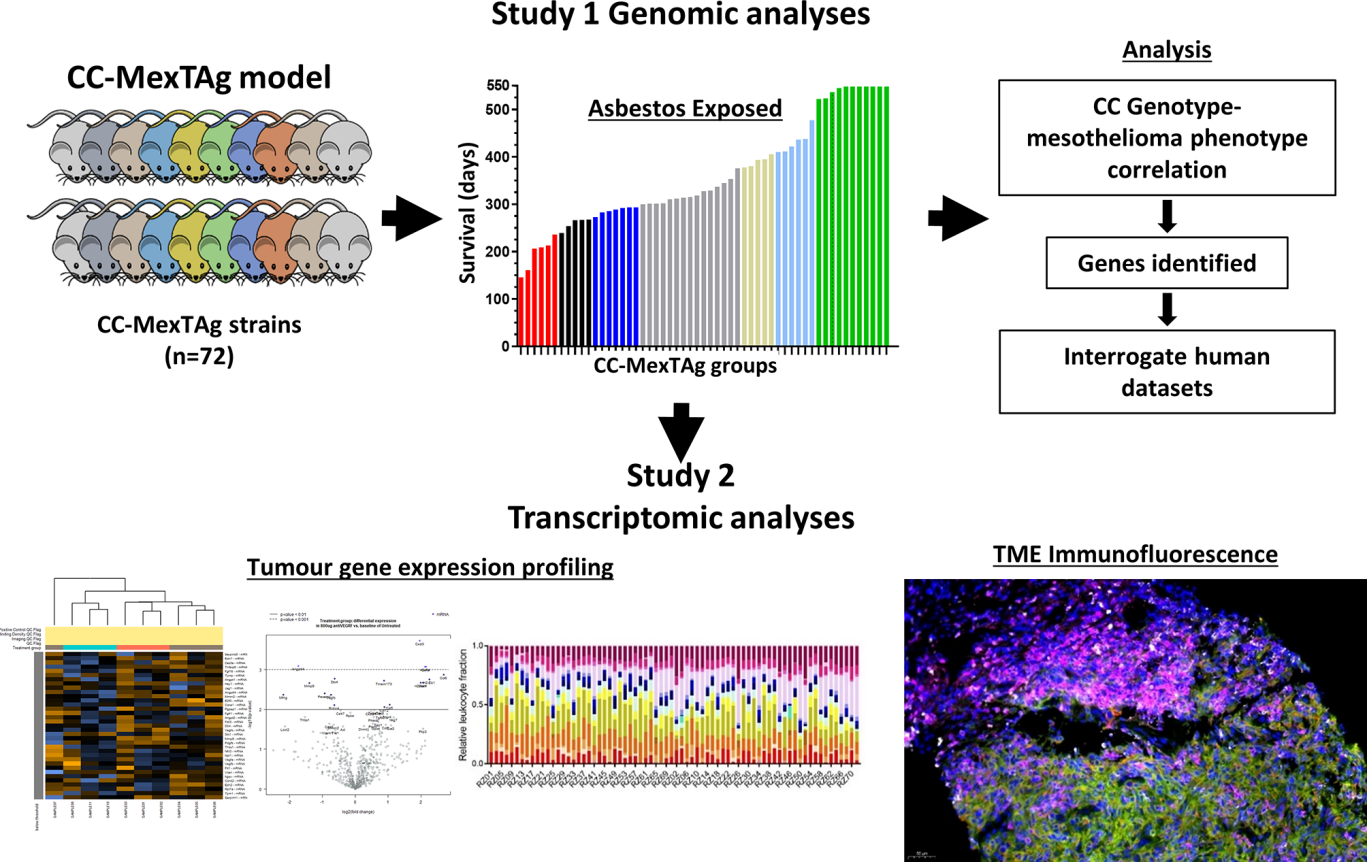
A schematic of the CC‐MexTAg experimental design. *The generation of CC‐MexTAg mice and their exposure to asbestos (Study 1):* Briefly, candidate modifier genes will be mapped with mesothelioma‐free survival, as a quantitative trait using the GeneMiner Bioinformatics pipeline. Genome wide scans will be used to define chromosomal locations of peak single nucleotide polymorphism (SNPs) associated with each of the characterized mesothelioma phenotypes, such as disease progression, latency, overall survival, and mesothelioma incidence. *Gene expression analysis and immunofluorescence analysis of tumors collected from CC‐MexTAg mice exposed to asbestos (Study 2):* Comprehensive analyses of gene expression profiles and immune cell infiltrate of the tumor microenvironment will be performed to identify any differences between distinct CC‐MexTAg groups. These data will be correlated back to phenotypic data from Study 1, to build a broader understanding of the impact of host genetics on asbestos related disease development.

Importantly, the development of the CC−MexTAg model has not only enabled data collection on numerous ARD phenotypic traits, but has enabled the generation of a large repository of tumor samples and tumor-derived cell lines, collected from animals that are either relatively resistant or highly sensitive to asbestos-induced cancer. Given recent insights provided by the CheckMate 743 study demonstrating for the first time first-line immune checkpoint blockade (nivolumab plus ipilimumab) provided a significant and clinically meaningful improvement in overall survival *versus* platinum plus pemetrexed chemotherapy for mesothelioma (118), this unique biological resource can be exploited for comprehensive genetic and immunohistological analysis on tumors collected from CC-MexTAg mice (**Figure 1**). The CCMT biobank will complement many of the recent ‘multi-omic’ informed human mesothelioma datasets, helping to overcome some of the limitations associated with conventional genetic studies aimed at identifying the role of host genetic factors associated with the development and immunological control of rare thoracic cancers like mesothelioma.

We believe this strategy will not only allow identification of host modifier genes associated with ARD development, but when correlated with data on immune microenvironment will help elucidate what is required to generate an effective immune response to asbestos induced cancers. In addition, our strategy provides a rational approach that could be applied to other thoracic cancers by taking advantage of the power of CC to define a protective host genetic background.

## Summary and Concluding Remarks

Thoracic cancers are a leading cause of death worldwide. While advances have been made in our understanding of how genetic alternations impact cancer development and treatment outcomes for common thoracic malignancies like lung cancer, our knowledge remains limited for less common cancers such as mesothelioma and TETs. Moreover, there is a paucity in our understanding of the complex biological interplay between the tumor and the immune microenvironment. Understanding how the host immune system interacts with a developing tumor is essential for the rational development of new or improved treatment regimens for thoracic malignancies. An often overlooked characteristic of the tumor host interaction, is the influence a hosts’ background genetics has on tumor development and how this affects treatment response. While previous conventional genomic studies are often limited to more common cancers, recent technical advances in computation biology, combined with the use of ‘system genetics’ approaches, now provide a framework for investigating more rare thoracic malignancies such as mesothelioma and TETs. To address these issues, we have developed the MexTAg Collaborative cross to identify host genes that affect asbestos-related disease. The CC-MexTAg mouse model embraces a systems genetics approach, linking the power of CC’s defined host background genetics, with gene expression analysis and unparalleled detailed spatial assessment of the immunological milieu of asbestos-induced mesothelioma. This unique model allows rapid identification of key host modifier genes and a comprehensive genomic and histopathological analyses of biological pathways associated with asbestos-induced mesothelioma development. These data can then be validated by interrogating the numerous data sets produced from current human genetic studies.

In conclusion, the CC-MexTAg mouse model, particularly in combination with contemporaneous tumor expression data, will provide a detailed picture of the role of modifier genes and their biological pathways associated with immune response in mesothelioma. Such data is essential to help identify potential druggable and translatable targets for the development of better treatment options and developing an effective anti-tumor immune response in malignant mesothelioma patients.

## Author Contributions

SF, KBe, SM and RK contributed to conception and design of the review. SF, KBu and KBe performed, collected and analyzed CC−MexTAg data. KBe and SF wrote the first draft of the manuscript. RK, SM, KBu, and GM wrote sections of the manuscript. All authors contributed to the article and approved the submitted version.

## Funding

The CC-MexTAg research program has been supported with funding from The US Department of Defense (CDMRP Ideas Award; CA170299), The National Health and Medical Research Council of Australia (APP1163861) and Insurance and Care NSW (icare).

## Conflict of Interest

The authors declare that the research was conducted in the absence of any commercial or financial relationships that could be construed as a potential conflict of interest.
